# Impact of Roux-en-Y Gastric Bypass on Mitochondrial Biogenesis and Dynamics in Leukocytes of Obese Women

**DOI:** 10.3390/antiox11071302

**Published:** 2022-06-29

**Authors:** Zaida Abad-Jiménez, Teresa Vezza, Sandra López-Domènech, Meylin Fernández-Reyes, Francisco Canet, Carlos Morillas, Segundo Ángel Gómez-Abril, Celia Bañuls, Víctor M. Víctor, Milagros Rocha

**Affiliations:** 1 Department of Endocrinology and Nutrition, University Hospital Doctor Peset, Foundation for the Promotion of Health and Biomedical Research in the Valencian Region (FISABIO), 46017 Valencia, Spain; zaiaji@alumni.uv.es (Z.A.-J.); vezza_ter@gva.es (T.V.); sandra.lopez@uv.es (S.L.-D.); meylyam05@gmail.com (M.F.-R.); francasu@alumni.uv.es (F.C.); carlos.morillas@uv.es (C.M.); celia.banuls@uv.es (C.B.); 2 Department of General and Digestive System Surgery, University Hospital Doctor Peset, Foundation for the Promotion of Health and Biomedical Research in the Valencian Region (FISABIO), 46017 Valencia, Spain; sean99cartu@yahoo.com; 3 Department of Surgery, Faculty of Medicine and Dentistry, University of Valencia, Av. Blasco Ibáñez 13, 46010 Valencia, Spain; 4 CIBERehd-Department of Pharmacology, University of Valencia, Av. Blasco Ibáñez 13, 46010 Valencia, Spain; 5 Department of Physiology, Faculty of Medicine and Dentistry, University of Valencia, 46010 Valencia, Spain

**Keywords:** bariatric surgery, obesity, mitochondrial dynamics, oxidative stress, inflammation

## Abstract

The chronic low-grade inflammation widely associated with obesity can lead to a prooxidant status that triggers mitochondrial dysfunction. To date, Roux-en-Y gastric bypass (RYGB) is considered the most effective strategy for obese patients. However, little is known about its molecular mechanisms. This interventional study aimed to investigate whether RYGB modulates oxidative stress, inflammation and mitochondrial dynamics in the leukocytes of 47 obese women at one year follow-up. We evaluated biochemical parameters and serum inflammatory cytokines -TNFα, IL6 and IL1β- to assess systemic status. Total superoxide production -dHe-, mitochondrial membrane potential -TMRM-, leucocyte protein expression of inflammation mediators -MCP1 and NF-kB-, antioxidant defence -GPX1-, mitochondrial regulation—PGC1α, TFAM, OXPHOS and MIEAP- and dynamics -MFN2, MNF1, OPA1, FIS1 and p-DRP1- were also determined. After RYGB, a significant reduction in superoxide and mitochondrial membrane potential was evident, while GPX1 content was significantly increased. Likewise, a marked upregulation of the transcription factors PGC1α and TFAM, complexes of the oxidative phosphorylation chain (I–V) and MIEAP and MFN1 was observed. We conclude that women undergoing RYGB benefit from an amelioration of their prooxidant and inflammatory status and an improvement in mitochondrial dynamics of their leukocytes, which is likely to have a positive effect on clinical outcome.

## 1. Introduction

Obesity is a complex and multifactorial disease currently affecting 650 million people worldwide and represents one of the largest problems facing public health in modern societies. Similar to other chronic diseases, it has been linked to several health complications, including dyslipidemia, hypertension, insulin resistance (IR), type 2 diabetes (T2D), heart disease and strokes, sleep apnoea and cancer, which are responsible for the significant morbidity and mortality associated with this global epidemic [1].

Lifestyle and pharmacological approaches are the most prescribed interventions to overcome this disease [2,3]. However, poor patient adherence can render these strategies ineffective insofar as achieving long-lasting benefits. In this sense, Roux-en-Y gastric bypass (RYGB) is a surgical weight loss treatment for morbidly and severely obese patients [4] that has consistent short- and long-term effects on many obesity hallmarks. [5,6]. Recent data indicate that, despite similar obesity rates among men and women, there is substantial gender disparity in the use of bariatric surgery, with women comprising 80% of the patients undergoing the procedure [7]. These differences may, in part, be due to gender-based differences in perceptions of obesity-related quality of life and body weight, which may affect the motivation for seeking surgery interventions and treatments.

Previous studies have highlighted the importance of reducing the inflammatory response and oxidative stress—mediated by increased antioxidant capacity and diminished levels of reactive oxygen species (ROS) production—as underlying mechanisms of the cardiometabolic changes associated with RYGB [6,8]. Mitochondria are known to play a key role in these processes, since not only are they the primary sources of cellular ROS, but their functions include ATP production by oxidative phosphorylation (OXPHOS), as well as regulation of calcium homeostasis and programmed cell death [9]. To deal with these challenges, mitochondria are amazingly plastic organelles that mediate a series of dynamic processes, such as mitochondrial fusion and fission, mitophagy and mitochondrial biogenesis, which determine mitochondrial morphology, quality and abundance.

There is growing evidence of an intriguing direct connection between mitochondrial dynamics and nutrient availability status [10], suggesting that modifications in mitochondrial architecture and networks are a mechanism of bioenergetic adaptation to metabolic demands. Interestingly, a reduction in the mitochondrial proteins mitofusin (MNF)1, MNF2, optic atrophy 1 (OPA1) and the dynamin-related protein 1 (DRP1) has been associated with an impairment of OXPHOS capacity, as well as defects in energy production in several metabolic diseases, including type 2 diabetes and obesity [11,12,13]. In line with this, a recent study reported that sedentary—and not active—subjects displayed an age-associated downregulation in MNF1, MNF2, DRP1 and OPA1, a process related to weight gain, muscle loss and inflammation [14].

In terms of metabolic demands, cellular starvation has been linked to the elongation of mitochondria through upregulated fusion [15] and increased ATP synthesis capacity [16], while a rich-nutrient environment tends to generate mitochondrial fragmentation [16,17] and apoptotic function via increased fission [18]. For its part, fragmentation probably reduces the mitochondrial oxidative metabolism, as displayed in the adipocytes of obese animal models and humans [19,20,21].

Despite enormous interest in the management of obesity, relatively little is known about the effects of RYGB surgery on the intricate biogenesis and function of mitochondria. RYGB has been reported to provide beneficial effects on the liver mitochondrial dynamics of diet-induced obese rats [22]; an increase in mitochondrial complexes I–V have been observed after surgery, as well as modifications in the expression of several mitochondrial proteins involved in mitophagy, mitochondrial biogenesis, fusion and fission. Moreover, consistent with previous studies performed on human adipose tissue [23] and skeletal muscle [24], RYGB showed an increase in the peroxisome proliferator-activated receptor gamma coactivator 1-alpha (PGC1α) [22], a key transcription factor regulating energy efficiency, as well as mitochondrial quality control and fatty acid oxidation [25]. Among the downstream mediators of PGC1α, the transcription factor A mitochondria (TFAM) is a transcription factor for mitochondrial DNA (mtDNA) implicated in mitochondrial-encoded gene transcription and mtDNA replication, whose expression is downregulated in the adipose tissue of obese patients [26]. In this line, myocytes derived from severely obese subjects undergoing RYGB show improved mitochondrial function in association with reduced Drp1 phosphorylation [27], while mitochondrial basal and maximal respiration rates in peripheral blood monocytes have been shown to increase rapidly after surgery [28].

However, to date, no studies have highlighted the effects of this surgical procedure on mitochondrial dynamics in human leukocytes. Therefore, based on the importance of reducing the obesity-associated inflammatory and prooxidative status, the present study aimed to explore whether RYGB-induced weight loss modulates fission and fusion proteins as well as overall mitochondrial quality control mechanisms in the leukocytes of obese women.

## 2. Materials and Methods

### 2.1. Subjects

The study cohort was composed of forty-seven obese patients undergoing RYGB, recruited at the Endocrinology and Nutrition Outpatient’s Department and the Department of General and Digestive System Surgery of the University Hospital Doctor Peset, Valencia (Spain). Recruitment was carried out from May 2017 to September 2019. All subjects agreed with the objectives and methodology of the study and gave their written informed consent to participate. The hospital’s Ethics Committee for Clinical Investigation approved the study (code 96/16), which was in line with the World Medical Association’s Declaration of Helsinki.

It is important to note that this study has arisen as part of a wider project, registered in clinicaltrials.gov under the study number NCT05071391. During the course of this project, we have revealed the implication of inflammation and oxidative stress in the pathogenesis of obesity and its comorbidities and have explored in depth the mechanisms associated with RYGB-induced weight loss [6,29].

Women aged from 18 to 65 years, with a body mass index (BMI) of ≥35 kg/m^2^ and assigned to RYBG intervention were eligible to be included in the study. Pregnancy or lactation, active infectious disease, thromboembolism, stroke or documented history of cardiovascular diseases, malignancies, severe renal or hepatic disease, drug abuse, chronic inflammatory disease and secondary obesity (hypothyroidism, Cushing’s syndrome) were established as exclusion criteria. We only targeted the female population to reduce potential bias due to gender confounding and interindividual variations.

### 2.2. Sample Collection, Anthropometric and Biochemical Determinations

The study cohort was composed of forty-seven obese patients undergoing RYGB, recruited at baseline and post-surgical (12 months after RYGB) appointments attended by subjects, during which blood samples were collected from the brachial vein under fasting conditions (10–12 h) between 8:00 and 9:30 a.m. Several anthropometric parameters were then measured as follows: systolic blood pressure (SBP) and diastolic blood pressure (DBP) were measured twice consecutively by an automatic sphygmomanometer; weight and height were measured with an electronic scale and stadiometer, respectively; BMI was calculated as weight (kg)/(height (m))^2^, and waist circumference was measured at the 10th rib and the iliac crest using a measuring tape. The percentage of excess weight loss (EWL) was calculated according to the formula [(preoperative weight−current weight)/(preoperative weight–ideal weight (considering BMI = 25 kg/m^2^))] × 100.

Levels of fasting glucose, total cholesterol, HDL cholesterol and triglycerides were obtained with a Beckman LX20 analyzer (Beckman Corp., Brea, CA, USA). Friedwald’s formula was employed to calculate LDL cholesterol. Insulin was measured with an immunoassay using the Architect Insulin Reagent Kit, and insulin resistance was estimated using the Homeostasis Model of Assessment (HOMA-IR = (fasting insulin (μU/mL) × fasting glucose (mg/dL)/405)). Glycated haemoglobin (HbA1c) was analysed employing an automated glycohemoglobin analyser (Arkray Inc., Kyoto, Japan). Serum levels of high sensitivity C-reactive protein (hsCRP) were analysed using an immunonephelometric assay (Behring Nephelometer II, Dade Behring, Inc., Newark, DE, USA) with an intra-assay coefficient of variation < 5.5%. Total leukocytes and neutrophils were determined in a COULTER^®^ LH 500 haematology blood analyser (Beckman Coulter Inc., Brea, CA, USA).

### 2.3. Isolation of Leukocytes

Blood collected in BD Vacutainer^®^ citrated tubes (approximately 15 mL) was mixed and incubated with dextran 3% for 45 min at room temperature (RT). The supernatant was placed over Ficoll-Paque Plus (GE Healthcare, Uppsala, Sweden) and centrifuged at 650× *g* for 25 min at RT. The resulting halo of peripheral blood mononuclear cells (PBMCs) was collected and centrifuged for 10 min at 650× *g*. The pellet of polymorphonuclear leukocytes (PMN) was incubated with a specific erythrocyte lysis buffer (Sigma-Aldrich, Inc., St. Louis, MO, USA) for 5 min. Finally, PBMCs and PMN pellets were washed twice in Hank’s Balanced Salt Solution (HBSS; Capricorn, Ebsdorfergrund, Germany) prior to the following experiments.

### 2.4. Protein Expression Analysis

PBMCs were lysed on ice for 15 min with RIPA Lysis Buffer supplemented with protease plus phosphatase inhibitors, and total protein concentration was quantified using the BCA assay (all reagents from Thermo Fisher Scientific, Waltham, MA, USA). Aliquots of 25 µg of protein were resolved on 8–16% or 4–20% gradient SDS-polyacrylamide gels (Invitrogen, Carlsbad, CA, USA) and then transferred to nitrocellulose membranes. Membranes were then blocked with 5% BSA or 5% skimmed milk in TBS-T for 1 h at RT with soft shaking. Proteins of interest were detected by incubating membranes overnight at 4 °C with the following primary antibodies: mouse monoclonal anti-OPA-1 (Ref. MABN737), rabbit polyclonal anti-MFN1 (Ref.ABC41), rabbit polyclonal anti-MFN2 (Ref. ABC42) and rabbit polyclonal anti-FIS-1 (Ref. ABC67) from Merck-Millipore (Burlington, MA, USA); mouse monoclonal anti-OXPHOS (Ref. ab110411), rabbit monoclonal anti-MIEAP (Ref. ab180154), rabbit polyclonal anti-PGC1ɑ (Ref. ab54481) and rabbit polyclonal anti-MCP-1 (Ref. ab73866) from Abcam (Cambridge, UK); rabbit monoclonal phospho-DRP1 (Ser^616^) from (Ref. 4494s) Cell Signalling Technology (Danvers, MA, USA); mouse monoclonal anti-mtTFA (Ref. sc-376672) from Santa Cruz (Dalas, TX, USA); mouse monoclonal anti-NF-кB (Ref. 33-9900) and rabbit polyclonal anti-GPX1 (Ref. PA5-30593) from Thermo Fisher Scientific (Waltham, MA, USA). Mouse monoclonal anti-actin (Ref. 3700T) from Cell Signalling Technology (Danvers, MA, USA), rabbit polyclonal anti-actin (Ref. A5060) from Sigma-Aldrich (San Luis, MO, USA) and mouse monoclonal anti-VDAC (Ref. ab14734) from Abcam (Cambridge, UK) were used as protein loading controls. The following day, membranes were incubated for 60 min at RT with the following secondary antibodies: goat anti-rabbit from Vector Laboratories (Ref. PI-1000-1) (Burlingame, CA, USA) and goat anti-mouse (Ref. 31430) from Thermo Fisher Scientific (Waltham, MA, USA). A summarised table (Table S1) of all antibodies used has been included as supplementary material. The chemiluminescence signal was detected with SuperSignal West Pico Plus or Femto from Thermo Fisher Scientific (Walthman, MA, USA) using the Fusion FX5 (Vilber Lourmat, Marne-La Vallée, France) imaging system. The quantification of protein levels was performed by densitometric analysis with Bio1D software v15.03a (Vilber Lourmat, Marne-La Vallée, France).

### 2.5. Evaluation of Systemic Cytokines TNFɑ, IL6 and IL1β

Blood in EDTA-coated tubes was used to obtain plasma samples by centrifugation (1500 g, 10 min, 4 °C). Serum concentrations of TNFɑ, IL6 and IL1β were analysed in duplicate with a Luminex^®^ 200 analyser system (Luminex Corporation, Austin, TX, USA) according to the Milliplex-Kit manufacturer’s procedure (Millipore Corporation, Billerica, MA, USA). Validation settings were intra- and inter-serial coefficient variations (CV) of <5.0% and <15.0%, respectively.

### 2.6. Superoxide Production and Mitochondrial Membrane Potential

The determination of superoxide production and mitochondrial membrane potential was assessed by static fluorometry using an IX81 Olympus fluorescence microscope coupled with the static cytometry software ScanR v2.03.2 (Olympus, Hamburg, Germany). In brief, 1.5 × 10^5^ PMN/wells were seeded in a 48-well plaque and incubated for 30 min at 37 °C with Dihydroethidium (DHE) and tetramethylrhodamine methyl ester (TMRM) probes for intracellular superoxide and mitochondrial membrane potential determination, respectively. Hoechst 33,342 was used to visualise cell nuclei. All fluorescent dyes were purchased from Life Technologies (Thermo Fisher Scientific, Waltham, MA, USA).

### 2.7. Statistical Analysis

This study was designed to achieve a power of 80% and detect significant (*p* < 0.05) differences of 20% in relation to the primary efficacy criterion—protein detection by Western blot—assuming a common SD of 25 units. Based on these premises, a minimum of 13 patients were required, as a loss-to-follow-up rate of 0% was estimated. SPSS 20.0 (IBM SPSS Statistic, Chicago, IL, USA) was employed to conduct the statistical analysis. Normality was checked by employing the Shapiro—Wilk test due to sample size. Parametric values are expressed as the mean ± standard deviation (SD) and non-parametric values as the median and interquartile range (25th–75th percentile). Qualitative data are expressed as percentages. Bar graphs were represented by the mean + standard error (SE). The paired Student’s *t*-test and Wilcoxon test were used to compare parametric and non-parametric data, respectively. Statistical significance was considered when *p* < 0.05 in all comparisons, with a confidence interval of 95%.

## 3. Results

This study was carried out on a cohort of 47 obese female patients with a mean age of 45.5 ± 10.2 years and a BMI of 40.3 ± 5.3 kg/m^2^. As expected, after RYGB surgery patients showed a considerable decrease in waist circumference (*p* < 0.001), BMI (*p* < 0.001), SBP and DBP (*p* < 0.01 for both) (Table 1).

Glucose metabolism parameters, such as HbA1c, insulin, glucose, and HOMA-IR (*p* < 0.001 for all), also improved. Similarly, triglycerides (*p* < 0.001), LDL cholesterol (*p* < 0.001) and total cholesterol (*p* < 0.001) showed a significant decrease, while HDL cholesterol levels (*p* < 0.001) had fallen one year after the intervention. Acute phase reactant hsCRP (*p* < 0.001) and total leukocyte count (*p* < 0.05) were lower after the intervention (Table 1).

**Figure 1:**
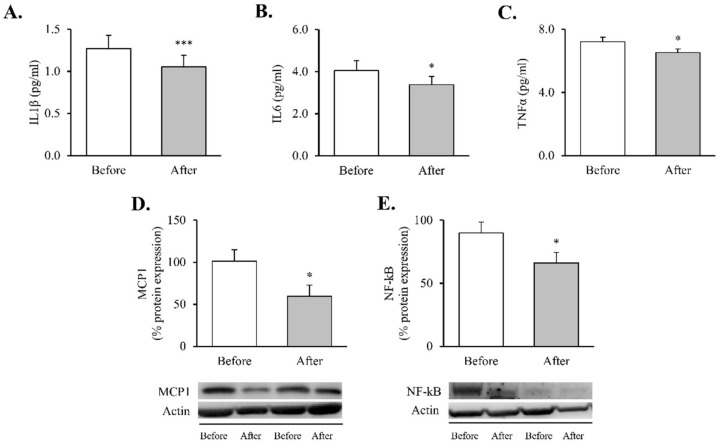
Proinflammatory markers in obese patients before and after RYGB. Serum levels of (**A**) TNFα (*n* = 32), (**B**) IL6 (*n* = 36) and (**C**) IL1β (*n* = 35). Leukocyte protein expression of inflammatory mediators and representative Western blot images of (**D**) MCP1 (*n* = 13) and (**E**) NF-kB (*n* = 21). Data are represented as the mean + SE. * *p* < 0.05, *** *p* < 0.001 when compared using a paired Student’s *t*-test. IL1β, interleukin 1β; IL6, interleukin 6; MCP1, monocyte chemoattractant protein 1, NF-κB, nuclear factor kB; RYGB: Roux-en-Y gastric bypass; TNFα, tumor necrosis factor alpha.

These changes were accompanied by reductions in systemic proinflammatory cytokines—TNFɑ (Figure 1A, *p* < 0.05), IL6 (Figure 1B, *p* < 0.05) and IL1β (Figure 1C, *p* < 0.001) —and were mirrored by a decline in intracellular mediators of inflammatory response in leukocytes—MCP1 and NF-κB proteins (Figure 1D,E, *p* < 0.05 for both).

**Figure 2:**
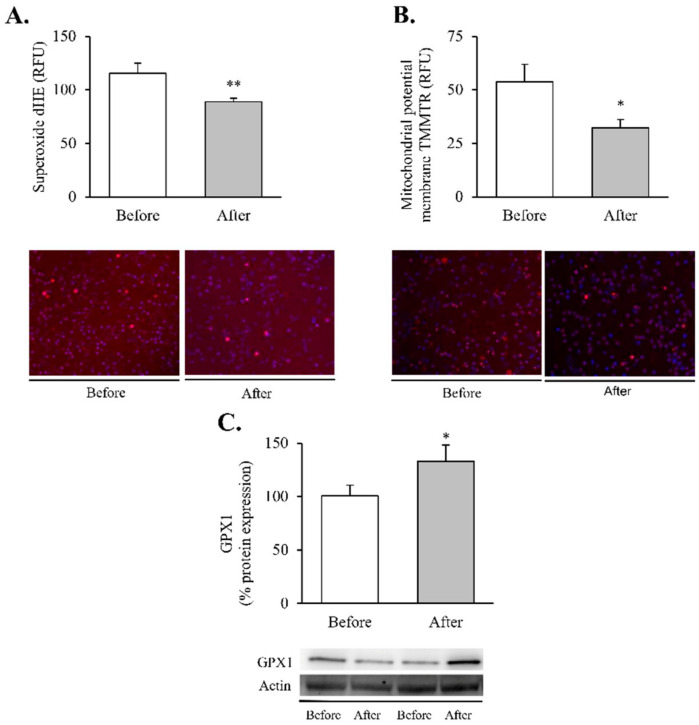
Oxidative stress and mitochondrial dysfunction markers in leukocytes of obese patients before and after RYGB. Evaluation of (**A**) total superoxide (*n* = 20) and (**B**) mitochondrial membrane potential (*n* = 21), expressed as arbitrary units of fluorescence and with representative images stained respectively with dHE (red) and TMRM (red), and Hoechst 33,342 for nuclei (blue). (**C**) Leukocyte protein expression of GPX1 and representative western blot images (*n* = 19). Data are represented as the mean + SE. * *p* < 0.05 ** *p* < 0.01 when compared using a paired Student’s *t*-test. dHE, Dihydroethidium; TMRM, ethyl ester of tetramethylrhodamine; GPX1, Glutathione peroxidase 1.

Since obesity-related inflammatory status has been closely linked to cell oxidative stress, we also aimed to analyse superoxide production, antioxidant defences and mitochondrial membrane potential (Figure 2) in leukocytes, widely known to be sensors of the whole-body’s responses to disease [30].

**Figure 3:**
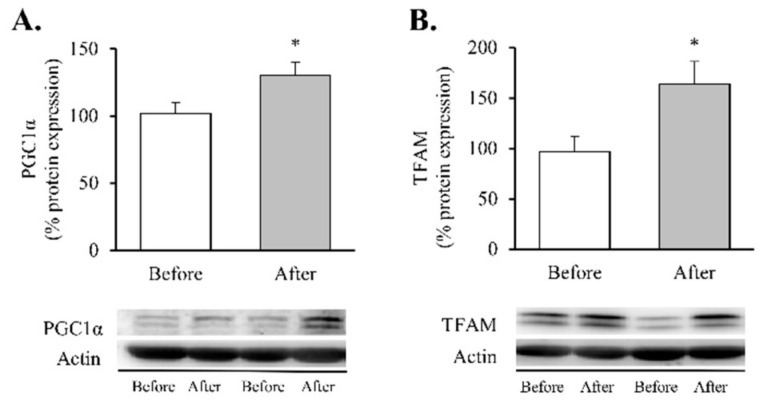
Transcription factors of mitochondrial biogenesis in leukocytes of obese patients before and after RYGB. Protein expression and representative Western blot images of (**A**) PGC1α (*n* = 10) and (**B**) TFAM (*n* = 16). Data are represented as the mean + SE. * *p* < 0.05 when compared using a paired Student’s *t*-test. PGC1α, Peroxisome proliferator-activated receptor γ co-activator 1α; TFAM, Transcription Factor A Mitochondrial.

Our findings showed a significant decrease in total superoxide (Figure 2A, *p* < 0.01) and mitochondrial membrane potential (Figure 2B, *p* < 0.05) and a restoration of the antioxidant enzyme GPX1 (Figure 2C, *p* < 0.05). Taken together, these results suggest a partial recovery of redox balance thanks to a decrease in ROS production and an increase in the antioxidant response. In line with these findings, and given the close relationship between obesity, oxidative stress and processes of mitochondrial dynamics, we decided to determine the impact of RYGB on several regulators and transcriptional coactivators of mitochondrial biogenesis in leukocytes of obese subjects before and after surgery (Figure 3). One year after the intervention, we observed a significant increase in the transcriptional coactivator PGC1α (Figure 3A, *p* < 0.05) and its downstream mediator TFAM (Figure 3B, *p* < 0.05), suggesting a mitochondrial network turnover.

**Figure 4:**
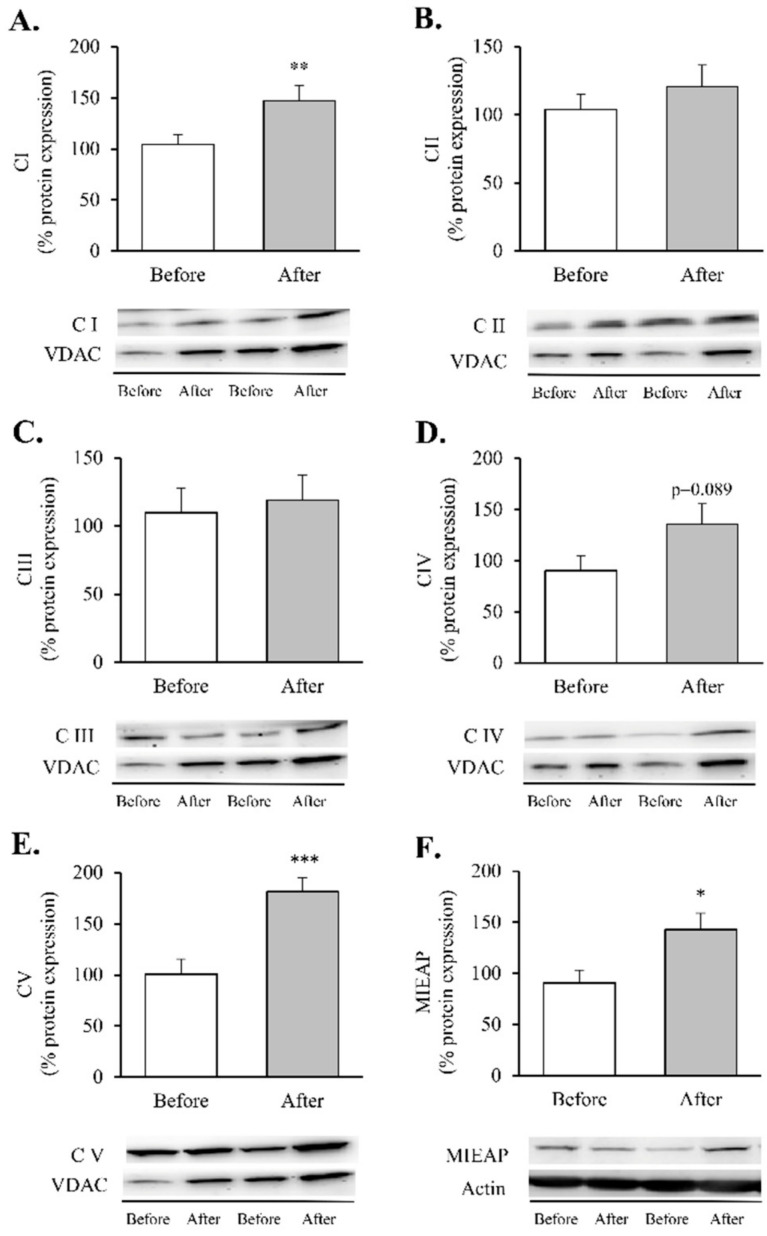
Mitochondrial complexes involved in the electron transport chain and MIEAP in leukocytes of obese patients before and after RYGB. Protein expression and representative Western blot images of (**A**) Mitochondrial complex I (*n* = 15), (**B**) Mitochondrial complex II (*n* = 15), (**C**) Mitochondrial complex III (*n* = 13), (**D**) Mitochondrial complex IV (*n* = 14), (**E**) Mitochondrial complex V (*n* = 14) and (**F**) MIEAP (*n* = 9). Data are represented as the mean + SE. * *p* < 0.05, ** *p* < 0.01, *** *p* < 0.001 when compared using a paired Student’s *t*-test. MIEAP, mitochondria-eating protein.

Our next step was to determine changes in the protein expression of the five mitochondrial OXPHOS complexes (Figure 4).

These assemblies provide most of the energy required for cellular function through an electrochemical proton gradient (or a proton motive force) between the mitochondrial matrix and the intermembrane space. After RYGB, our patients showed an increase in complexes I and V (Figure 4A,E, *p* < 0.05 and *p* < 0.001, respectively) and an upward trend in complex IV (Figure 4D, *p* = 0.089), which was also accompanied by a substantial upregulation of MIEAP (Figure 4F, *p* < 0.05).

**Figure 5:**
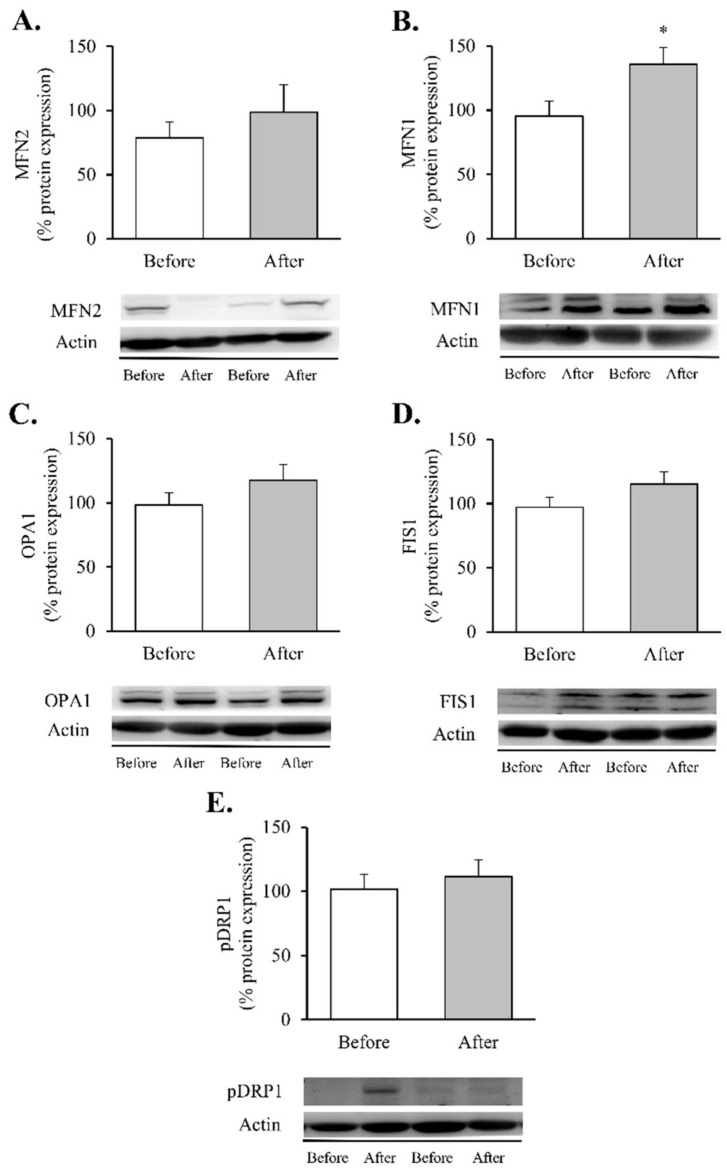
Mitochondrial fusion and fission markers in leukocytes of obese patients before and after RYGB. Leukocyte protein expression and representative Western blot images of (**A**) MFN2 (*n* = 12), (**B**) MNF1 (*n* = 13), (**C**) OPA1 (*n* = 14), (**D**) FIS1 (*n* = 16) and (**E**) p-DRP1 (*n* = 14). Data are represented as the mean + SE. * *p* < 0.05 when compared using a paired Student’s *t*-test. FIS1, mitochondrial fission 1; MNF1, mitofusin 1; MFN2, mitofusin 2; OPA1, optic atrophy protein 1; p-DRP1, dynamin-related protein 1.

Finally, associated with these changes, we detected intriguing alterations in mitochondrial dynamics post-surgery (Figure 5).

Although the increase in MNF2 (Figure 5A) and OPA1 did not reach statistical significance (Figure 5C), a significant increase in MFN1 protein content (Figure 5B, *p* < 0.05) was observed in leukocytes after RYGB. In contrast, the fission proteins FIS1 and p-DRP1 (Ser^616^) remained unchanged (Figure 5D,E, respectively). As a whole, these results lead us to hypothesise that RYGB restores mitochondrial homeostasis by reducing the inflammatory response and oxidative stress parameters and by modulating mitochondrial dynamics through activation of transcriptional factors involved in the synthesis and degradation of mitochondrial components, OXPHOS complexes and fusion and fission processes, though this requires further confirmation.

## 4. Discussion

In the present study, we have seen how obese women undergoing RYGB exhibited improvements in several clinical and metabolic outcomes, including sustained weight loss, enhanced glucose homeostasis and lipid profile, as well as a reduction in systemic inflammatory parameters. These improvements were accompanied by a reduction in intracellular inflammatory pathways in leukocytes and a slowing down of oxidative stress. Interestingly, our findings revealed that RYGB regulates several processes of mitochondrial dynamics, including fusion/fission, the repair or removal of dysfunctional organelles and mechanisms of mitochondrial biogenesis. Altogether, these findings represent novel and relevant evidence of the physiological and molecular mechanisms involved in the beneficial effects of this surgical procedure in obesity and its associated metabolic comorbidities.

The pathophysiological mechanisms underlying the relationship between obesity and metabolic dysfunction are likely to be multifactorial. Over the last few years, bariatric surgery has proven to be successful in treating morbid/severe obesity, improving patient quality of life and providing durable and effective results with respect to various metabolic parameters—namely, weight loss, improvement of lipid profile, reduction of cardiovascular risk and glycaemic control [31,32,33,34,35], which our findings affirm. It is worth noting that inflammation is reported to be the main player linking obesity with its related metabolic perturbations [36] and is ameliorated after weight loss. A significant reduction in systemic cytokines—TNFɑ, IL6 and IL1β—and decreased MCP1 and NF-κB protein expression were detected in the leukocytes of our patients after RYGB, which is in line with previous reports of an overall reduction in inflammatory markers in leukocytes [37] and adipose tissue [23]. The complex inflammatory network that characterises obesity is mostly activated by an oxidative stress status. Indeed, we have previously reported that increasing fat accumulation leads to both excessive ROS release and mitochondrial dysfunction in peripheral leukocytes [38]. Of note, and consistent with our previous study carried out in both sexes [6], an amelioration of oxidative stress parameters was observed after bariatric surgery. In this sense, Monzo-Beltran et al. also reported an adaptive antioxidant response of leukocytes after RYGB, manifested by higher intracellular SOD1, GPX1 and catalase activity [39].

Mitochondrial biogenesis is a self-renewal route by which new mitochondria originate from those that already exist in order to reduce mitochondrial dysfunction, which is regulated by AMP-activated protein kinase (AMPK), a major energy sensor of the cell [40]. Specifically, alterations in cellular energy consumption, energy production and AMP/ATP ratio can lead to the activation of this Ser/Thr kinase. In such a scenario, AMPK shuts down energy-consuming anabolic systems while switching on catabolic pathways to generate ATP [40]. Simultaneously, the kinase downregulates the expression of lipid synthesis genes while enhancing the expression of genes associated with glycolysis, glucose transport and mitochondrial activity [41,42,43]. Regarding this last point, it is important to note that the expression of genes involved in either mitochondrial activity or lipid oxidation in skeletal muscle was induced in transgenic mice overexpressing an activated form of the AMPK-γ-3 subunit. [44,45]. In contrast, increased mitochondrial respiration and biogenesis in response to energy deprivation was not observed in mice expressing a dominant negative form of AMPK [46]. In this way, these studies, and others [47], have identified AMPK as an essential regulator of mitochondrial biogenesis. Interestingly, evidence suggests that AMPK activity is reduced in obesity, suggesting it may be a therapeutic target [48]. In this regard, we have recently reported an upregulation of this kinase after RYGB surgery [29]. This tightly coordinated process implicates several transcriptional regulators (mainly PGC1α, NRF1 and NRF2) that activate TFAM [49], thus, leading to mitochondrial transcription and mitochondrial genome replication and, in turn, to the generation of new organelles. Among these intricate transcriptional regulators, PGC1α has attracted great attention within the field of obesity research due to its essential role in regulating the efficiency of energy metabolism, as well as in mitochondrial quality control and fatty acid oxidation [25,50]. Its expression has been found to be reduced in the adipose tissue of obese humans [26,51], while it is upregulated after weight loss induced by RYGB [23,24], thus, explaining the increase in PGC1α we observed in leukocytes at one-year follow-up. Studies have suggested that these changes were associated with a significant increase in TFAM, a key regulator of mtDNA replication. TFAM deletion in mutant mice induces obesity and diabetes [52], probably due to a remodelling of the respiratory chain through the downregulation of multiple proteins involved in oxidative phosphorylation.

Alterations of mitochondrial content and activity have emerged as critical features of obese in rodents [53] and humans [54,55]. In fact, OXPHOS failure can lead to ROS overproduction and accumulation of unhealthy mitochondria [56], which need to be repaired or eliminated to preserve the stability and health of cells [57]. Recently, a novel mechanism has been proposed for mitochondrial quality control, in which the regulator MIEAP induces intramitochondrial structures that engulf and degrade damaged mitochondria by the accumulation of lysosomes [58].

Our results suggest that RYGB drives an upregulation of levels of the OXPHOS proteins CI and CV (initial and final complexes in the electron transport chain), along with an increase in MIEAP. This points to a post-surgical amelioration of mitochondrial activity and function that could be associated with the improvement in metabolic outcomes observed in our cohort. Previous studies focused on different target tissues (liver, adipose or muscle) and models (cellular or animal) have highlighted the upregulation of OXPHOS complex expression upon caloric restriction [59] or RYGB [22,60,61,62]. In accordance with Nijhawan et al., who reported that mitochondrial basal and maximal respiration rates in peripheral blood monocytes increased rapidly after surgery [28], we highlight the effects of this surgical procedure on the mitochondrial dynamics of human leukocytes from obese women. It is relevant to note that mitochondrial quality control also implies events of fusion and fission, by which cells mediate morphological plasticity and regulate energy expenditure and bioenergetic efficiency. These processes are in turn mediated by large guanosine triphosphatases (GTPases), including MNF1, MTF2, OPA1, DRP1 and FIS1 [63,64], which help to balance the fusion and division of the two lipid bilayers that surround mitochondria. In line with these findings, previous studies performed on obese humans have revealed a direct relationship between mitochondrial dynamics and the balance of nutrient supply/energy demand, suggesting that the remodelling of mitochondrial morphology and networks constitutes bioenergetic adaptation to metabolic requests [18,65]. In particular, several authors have argued that obesity is characterised by a reduced gene expression of OPA1 and MFN1 in rat liver and skeletal muscle, which may contribute to mitochondrial dysfunction [66]. Decreased MFN1 and MFN2 and increased DRP1 have also been reported in the skeletal muscle of obese patients [65,67,68,69].

Although there is increasing evidence that RYGB can improve mitochondrial fusion [70,71], data are limited and controversial. Saks et al. observed significant increases in the expression of hepatic MFN1 and OPA1 in the liver of obese rats following RYGB surgery [22], while, more recently, Kugler et al. found no changes in these proteins when analysed in myotubes derived from severely obese individuals seven months after RYGB [27]. In the case of mitochondrial fission proteins, there is controversy surrounding the effects of RYGB on FIS1 and DRP1 protein levels [22,27,69], which calls for future investigation. Our findings bring the knowledge a step further by illustrating how bariatric surgery influences mitochondrial dynamics in leukocytes of obese humans through an increase in MFN1. In contrast, the changes we observed in MNF2, OPA1, DRP1 or FIS1 did not reach statistical significance, which points to a tissue-specific regulation. This boosted mitochondrial fusion might contribute positively to the enlargement and functionality of the mitochondrial network by stimulating OXPHOS expression and respiration, as reported in the liver of obese rats [22].

The strengths of our study include its design, based on a population of women with obesity, which allows an accurate comparison of the implications of weight loss. We have evaluated intracellular responses to weight loss following RYGB, thus, representing, as far as we know, the first study to address changes in mitochondrial dynamics in the leukocytes of obese women. However, the present study has some limitations, including the relatively small size of the study population, though we would like to point out that our data are supported by sample size calculation. Additionally, we notice the impossibility of demonstrating a causal relationship between the improvement in metabolic outcomes induced by RYGB and the modulation of mitochondrial dynamics processes. Therefore, future randomised investigations are needed to determine the mechanism underlying the metabolic improvement detected in women after RYGB-induced weight loss. Such studies will undoubtedly constitute an important step toward developing strategies for preventing and treating obesity.

## 5. Conclusions

The present study endorses bariatric surgery as a novel strategy that improves the clinical hallmarks of obesity and its related comorbidities. Our findings suggest that women undergoing RYGB benefit from an amelioration of their prooxidant and inflammatory status and enhanced mitochondrial dynamics in their leukocytes, which could be responsible for the overall improvement in anthropometric and clinical features reported after surgery.

Given the essential role of mitochondria in energy metabolism, a better understanding of the molecular mechanisms that underlie mitochondrial dynamics may help to identify therapeutic targets to prevent and treat numerous diseases based on mitochondrial dysfunction, such as obesity and its associated comorbidities.

## Figures and Tables

**Table 1:** Clinical features of the study population before and after RYGB.

Parameters	Before	After
n (females %)	47 (100)	
Age (years)	45.5 ± 10.2	
Weight (kg)	107.1 ± 15.6	76.3 ± 12.0 ***
BMI (kg/m^2^)	40.3 ± 5.3	29.0 ± 4.3 ***
EWL (%)		81.1 ± 29.7
Waist (cm)	114.3 ± 10.5	88.8 ± 12.0 ***
SBP (mmHg)	130.6 ± 16.1	121.6 ± 18.3 **
DBP (mmHg)	80.6 ± 10.2	73.5 ± 11.0 **
Glucose (mg/dL)	96.4 ± 12.5	84.0 ± 6.9 ***
Insulin (μU/mL)	14.4 ± 7.6	7.0 ± 3.1 ***
HOMA-IR	3.52 ± 2.18	1.44 ± 0.72 ***
HbA1c (%)	5.47 ± 0.54	5.16 ± 0.35 ***
TC (mg/dL)	190.0 ± 32.6	169.8 ± 26.2 ***
HDLc (mg/dL)	48.1 ± 8.1	59.5 ± 9.6 ***
LDLc (mg/dL)	125.1 ± 40.2	97.2 ± 21.0 ***
TG (mg/dL)	95.5 (73.8, 136.5)	76.0 (56.0, 100.5) ***
hsCRP (mg/L)	4.69 (2.08, 8.29)	0.79 (0.28, 1.48) ***
Leukocytes (10^3^/μL)	7.74 ± 2.41	6.39 ± 1.98 *
**Treatment**		
Hypertension % (n)	36.2 (17)	14.9 (7)
Hyperlipidemia % (n)	21.3 (10)	10.6 (5)
T2D % (n)	27.7 (13)	0 (0)

Data are expressed as mean ± SD or percentage (n). TG and hsCRP are represented as median and IQ range (25% and 75% percentile). Values were statistically compared with a paired Student’s *t*-test or Wilcoxon test and were considered significant when * *p* < 0.05, ** *p* < 0.01 and *** *p* < 0.001. BMI, Body mass index; DBP, Diastolic blood pressure; EWL, Excess weight loss; HbA1c, Glycated haemoglobin; HDLc, HDL cholesterol; hsCRP, High sensitive C-reactive protein; IL1β, Interleukin 1β; IL6, Interleukin 6; LDLc, LDL cholesterol; SBP, Systolic blood pressure; TC, Total cholesterol; TG, Triglycerides; T2D, Type 2 diabetes.

## Data Availability

The data presented in this study are available upon request from the corresponding authors. The data are not publicly available due to ethical reasons.

## Supplementary Materials

The following are available online at https://www.mdpi.com/article/10.3390/antiox11071302/s1, Table S1: Detailed information of all the antibodies used.
