# Supplementary material for: Impact of Roux-en-Y Gastric Bypass on Mitochondrial Biogenesis and Dynamics in Leukocytes of Obese Women
Source: Antioxidants (Basel). 2022 Jun 29;11(7):1302. doi: 10.3390/antiox11071302 (PMC9312345; doi:10.3390/antiox11071302)
Supplement: Supplementary file 1 [file antioxidants-11-01302-s001.zip › antioxidants-1750880-supplementary.pdf]

Table S1: Detailed information of all the antibodies used.

Protein	Clone	Peptide / Inmunogen
**OPA1**	1OPA-1A8	Recombinant protein corresponding to mouse OPA1
**MFN1**	Polyclonal	KLH-conjugated linear peptide corresponding to the cytoplasmic domain of human Mitofusin-1
**MNF2**	Polyclonal	KLH-conjugated linear peptide corresponding to the cytoplasmic domain of human Mitofusin-2
**FIS1**	Polyclonal	KLH-conjugated-linear peptide corresponding to the cytoplasmic topological domain of human Mitochondrial fission 1 protein.
**OXPHOS**	Monoclonal	NA
**MIEAP**	EPR13704	Recombinant fragment within Human Spata18 aa 100-250
**PGC1a**	Polyclonal	Synthetic peptide corresponding to Human PGC1 alpha + beta aa 750-850
**MCP1**	Polyclonal	NA
**pDRP1**	Monoclonal	NA
**mtTFA**	Monoclonal	NA
**NFKB**	2A12A7	Recombinant protein (~175 amino acids) from the C-terminal portion of human NF-kappa-B (p65)
**GPX1**	Polyclonal	Synthetic peptide corresponding to a region within amino acids 127 and 203 of Human GPX1
**Actin Rb**	Polyclonal	N-terminal actin peptide attached to a multiple antigen peptide (MAP) backbone.
**Actin Ms**	Monoclonal antibody is produced by immunizing animals with a synthetic peptide corresponding to amino-terminal residues of human β-actin.	NA
VDAC	20B12AF2	Recombinant full length protein. This information is proprietary to Abcam and/or its suppliers.

NA, not available
